# Gazer-Theta: LLVM-based Verifier Portfolio with BMC/CEGAR (Competition Contribution)

**DOI:** 10.1007/978-3-030-72013-1_27

**Published:** 2021-02-26

**Authors:** Zsófia Ádám, Gyula Sallai, Ákos Hajdu

**Affiliations:** 8 Eindhoven University of Technology, Eindhoven, The Netherlands; 9 Aalborg University, Aalborg East, Denmark; 10 Budapest University of Technology and Economics, Budapest, Hungary; 11 SonarSource S.A., Geneva, Switzerland

## Abstract

Gazer-Theta is a software model checking toolchain including various analyses for state reachability. The frontend, namely Gazer, supports C programs through an LLVM-based transformation and optimization pipeline. Gazer includes an integrated bounded model checker (BMC) and can also employ the Theta backend, a generic verification framework based on abstraction-refinement (CEGAR). On SV-COMP 2021, a portfolio of BMC, explicit-value analysis, and predicate abstraction is applied sequentially in this order.

## 1 Verification Approach and Software Architecture

GAZER-THETA is a software model checking toolchain with two main components: GAZER, an LLVM-based frontend and THETA, a generic model checking framework. An overview of the architecture and the verification approach can be seen in Figure 1.

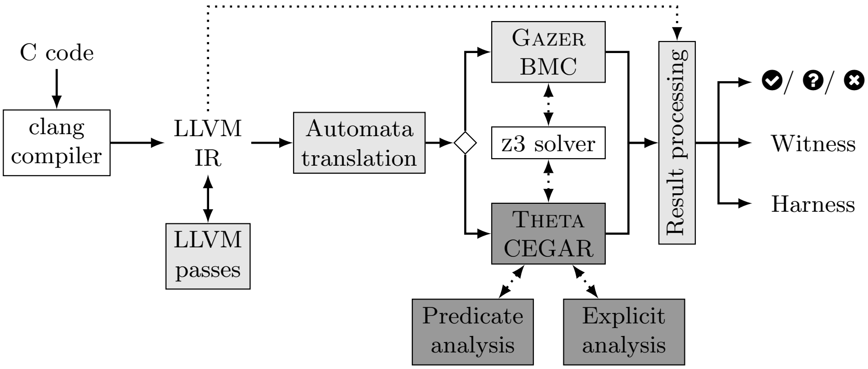

**Fig. 1.** Overview of the architecture. Solid arrows represent the workflow, dashed arrows indicate dependency. GAZER and THETA components are denoted by lighter and darker backgrounds, respectively.

* Jury member representing GAZER-THETA at SV-COMP 2021.

*Gazer.* Gazer [7] is a verification frontend for C programs written in C++17, using the LLVM compiler infrastructure.[3] The input is a C program (possibly consisting of multiple source files) that is first translated to the LLVM IR (intermediate representation) using the *clang* compiler. Next, various built-in and custom *LLVM passes* are executed to perform optimizations (e.g., inlining, constant propagation, assertion lifting) and transformations (e.g., adding traceability information) on the IR. The LLVM IR is then transformed into different variants of *control flow automata* (CFA), depending on the backend to be used. Gazer includes a built-in variant [5,7] of *bounded model checking* [2], relying on the z3 SMT solver [6]. The other supported backend is Theta (to be presented below). Currently, both backends provide analysis for *reachability properties*.

In the final step, the "raw" results of the backends are processed to produce a verdict (safe, unsafe, unknown) and a witness. Currently, Gazer only supports violation witnesses, both in a user-friendly syntax and in the format of SV-COMP. Furthermore, Gazer is also capable of generating executable test harnesses that can be used, e.g., in a debugger to reach the property violation.

*Theta.* Theta [8] is a generic and modular model checking framework written in Java 11, providing abstraction- and CEGAR-based analyses [4] for various formalisms, including CFA. Theta is highly configurable, supporting different abstract domains (such as *explicit-value analysis* [1] or *predicate abstraction* [3]) and refinement strategies, mostly based on interpolation (using SMT solvers such as z3 [6]). In the explicit-value analysis, only a subset of program variables is tracked, while predicate abstraction keeps track of logical facts and relationships instead of concrete values.

*Verification portfolio.* Based on our preliminary experiments, at SV-COMP 2021, we apply a sequential portfolio consisting of 3 steps, as illustrated by Figure 2. The portfolio is implemented as a Python script, which calls the tools described previously. First, bounded model checking is performed with a 150s time limit, which – in our experience – can already solve many unsafe instances. If BMC is inconclusive, we move on to an explicit-value analysis with a 100s limit, which can be effective for simpler, mostly deterministic programs. Finally, if the result is still unknown, we move on to the more heavyweight method of predicate abstraction. If any of the phases reports an unsafe result, as an additional step, we generate an executable test harness from the counterexample and check if the program actually reaches the property violation. This allows us to filter out some false positives (by reporting unknown instead of unsafe).

## 2 Strengths and Weaknesses

Gazer-Theta currently targets reachability analysis so we participate in the *ReachSafety* category, excluding subcategories *Arrays*, *Heap* and *Sequentialized*, due to features with limited support (e.g., pointers). The strength of the tool is

[3] https://llvm.org/

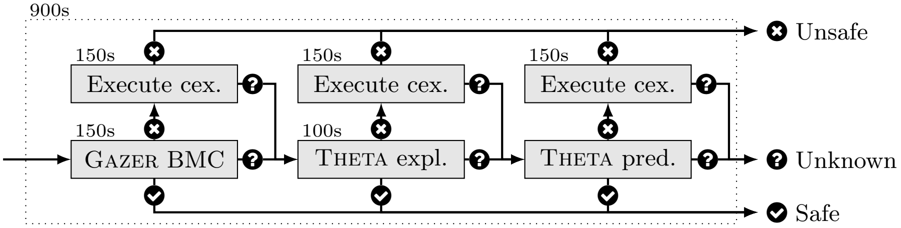

**Fig. 2.** Overview of the portfolio approach. Symbols ✔, ?, ✖ indicate safe, inconclusive and unsafe results, respectively. Numbers indicate the time limit of each phase.

its modularity and configurability, combining the advantages of different analyses into a diverse portfolio. Out of the 3679 tasks, there are 1722 confirmed correct (1079 safe, 643 unsafe), 4 unconfirmed correct, and 13 incorrect (false positive) results. A majority of the solved tasks (86% of 1722) come from the BMC phase; with a few exceptions, the CEGAR analyses need to be utilized only for safe instances (though they could also handle most of the tasks solved by BMC based on our experiments). The explicit-value analysis handles further 100 tasks in the *ECA* subcategory, while predicate abstraction solves 130 additional instances from *Loops* and *ProductLines*. Surprisingly, BMC can actually solve a significant amount (857) of safe instances as well, which can be attributed to LLVM optimizations and enhancements in the algorithm [7]. Furthermore, we also observed that executable harnesses could rule out many (142) false positives.

The weakness of Gazer-Theta is its limited support for certain features, such as arrays, bit-precise reasoning (only available for BMC), and pointers. We also observed that the LLVM IR representation often results in large CFA (e.g., many temporary variables due to SSA form), which makes reasoning harder via CEGAR (as witnessed, e.g., by the *ECA* subcategory). Currently, the tool gives empty correctness witnesses only meeting syntactical requirements, but surprisingly most of them were accepted. Furthermore, our violation witnesses are quite "sparse" due to heavy usage of optimization passes, but some validators can still prove their correctness. The 13 false positive results are caused by unsupported library functions (related to floats) treated as external calls with undefined (arbitrary) behavior.

## 3 Tool Setup and Configuration

The competition contribution is based on Gazer v1.2.1[4] and Theta v2.5.0.[5] Additionally, the BMC backend of Gazer uses z3 version 4.8.6, while Theta is based on z3 version 4.5.0. The projects' repositories contain instructions on building the tools, but an archive can be found on Zenodo[6] with pre-built binaries

4 https://github.com/ftsrg/gazer/releases/tag/v1.2.1
5 https://github.com/ftsrg/theta/releases/tag/v2.5.0
6 http://doi.org/10.5281/zenodo.4483627

for Ubuntu 18.04 or 20.04. The toolchain requires packages `clang-9`, `libgomp1`, `llvm-9`, `openjdk-11-jre-headless` and `python3` to be installed. The entry point of the toolchain is `scripts/gazer_starter.py`, which takes the verification task (C program) as its only mandatory input and runs the portfolio. No other parameters or configuration is required. Optionally, the output directory can be set (`--output`) and the version can be queried (`--version`).

## 4 Software Project

Gazer and Theta are maintained by the Critical Systems Research Group[7] of the Budapest University of Technology and Economics with various contributors. The projects are available open-source on GitHub[8] under an Apache 2.0 license.

*Acknowledgment.* The authors would like to thank Tamás Tóth, László Radnai, Mihály Dobos-Kovács, István Majzik, Zoltán Micskei, András Vörös and Vince Molnár for their contributions to the projects; and the competition organizers, especially Dirk Beyer for their help during the preparation for SV-COMP.

This research has received funding from the EU ECSEL JU under the H2020 Framework Programme, JU grant nr. 826452 (Arrowhead Tools project) and from the partners' national funding authorities.

## References

[1] Beyer, D., Löwe, S.: Explicit-state software model checking based on CEGAR and interpolation. In: FASE 2013, LNCS, vol. 7793, pp. 146–162. Springer (2013). 10.1007/978-3-642-37057-1_11

[2] Biere, A., Cimatti, A., Clarke, E.M., Zhu, Y.: Symbolic model checking without BDDs. In: TACAS 1999, LNCS, vol. 1579, pp. 193–207. Springer (1999). 10.1007/3-540-49059-0_14

[3] Graf, S., Saidi, H.: Construction of abstract state graphs with PVS. In: CAV1997, LNCS, vol. 1254, pp. 72–83. Springer (1997). 10.1007/3-540-63166-6_10

[4] Hajdu, Á., Micskei, Z.: Efficient strategies for CEGAR-based model checking. Journal of Automated Reasoning **64**(6), 1051–1091 (2020). 10.1007/s10817-019-09535-x

[5] Lal, A., Qadeer, S., Lahiri, S.: Corral: A solver for reachability modulo theories. In: CAV 2012. LNCS, vol. 7358, pp. 427–443. Springer (2012). 10.1007/978-3-642-31424-7_32

[6] de Moura, L., Bjørner, N.: Z3: An efficient SMT solver. In: TACAS 2008, LNCS, vol. 4963, pp. 337–340. Springer (2008). 10.1007/978-3-540-78800-3_24

[7] Sallai, Gy.: LLVM IR-based Transformations for Software Model Checking. Master’s thesis, Budapest University of Technology and Economics (2019)

[8] Tóth, T., Hajdu, Á., Vörös, A., Micskei, Z., Majzik, I.: Theta: a framework for abstraction refinement-based model checking. In: FMCAD 2017. pp. 176–179 (2017). 10.23919/FMCAD.2017.8102257

